# Liquid biopsy: expanding the frontier of circulating biomarker discovery and validation in breast cancer

**DOI:** 10.20517/cdr.2019.99

**Published:** 2019-12-19

**Authors:** Philip C. Miller, Dorraya El-Ashry, Marc E. Lippman

**Affiliations:** ^1^Department of Oncology, Lombardi Comprehensive Cancer Center, Georgetown University School of Medicine, Washington DC, WA 20007, USA.; ^2^Breast Cancer Research Foundation, New York, NY 10036, USA.; ^3^Department of Laboratory Medicine and Pathology, Masonic Cancer Center, University of Minnesota, Minneapolis, MN 55455, USA.

**Keywords:** Liquid biopsy, breast cancer, cell-free tumor DNA, CTC, cancer associated fibroblast

## Abstract

Liquid biopsies represent an attractive, minimally-invasive alternative to surgical sampling or complex imaging of breast cancer and breast cancer metastasis. Here we present a summary of the major biomarker components often evaluated in liquid biopsy samples from patients with breast cancer, including circulating tumor cells, circulating cell-free tumor DNA, and cancer-associated plasma proteins. We discuss recent advancements in methods of detection and use of these biomarkers in breast cancer. Finally, we highlight some of our own recent contributions to breast cancer liquid biopsy, including the identification and characterization of circulating Cancer Associated Fibroblasts.

## Introduction

Since the first reported observation of circulating tumor cells (CTCs) by Ashworth^[1]^ in 1869, there has been significant interest in assessing CTCs and other circulating biomarkers in cancer patients, their associations with disease state and prognosis, and their potential value as predictors of treatment response or tools to monitor disease progression. The idea of a “liquid biopsy” that enables the assessment of informative biomarkers through minimally invasive means is a concept that is widely supported among patients, clinicians, and researchers. Currently, the biomarkers most commonly evaluated in liquid biopsy studies are CTCs, circulating cell-free tumor DNA (ctDNA), and protein biomarkers found in plasma or serum. In the last decade, many competing and complementary technologies and methods have been developed for the detection of cancer associated markers in circulation. However, it remains a challenge to identify the best way to use these markers as tools to help guide breast cancer therapy. A major challenge in the treatment of breast cancer is the emergence of therapeutic resistance. The ability to monitor breast cancer responses to therapy in real-time to determine the need for alternative treatment in the event that a tumor develops resistance would revolutionize breast cancer care. Currently, studies on long-term treatment efficacy are limited by the need to monitor actual recurrent breast cancer. Liquid biopsy boasts the potential to follow one or more circulating biomarkers in real time as a surrogate for therapeutic success or failure. Our recent collaborative research efforts have focused on the discovery and validation of circulating cancer associated fibroblasts (cCAFs) as a novel cancer biomarker, and the use of liquid biopsy to monitor levels of cCAFs and other circulating biomarkers associated with breast cancer stage, metastasis, survival, and therapeutic response.

## Circulating cell-free tumor DNA is a prognostic and predictive marker of breast cancer

Recent developments in genomic technology enable the large-scale use of massively parallel DNA sequencing to detect genomic alterations in tumor samples. Targeted mutation panels (such as the FoundationOne® Liquid or Guardant360® tests) identify mutations in commonly mutated cancer-driver genes, which can be used to monitor treatment response or progression of many tumor types. ctDNA consists of small fragments of tumor DNA found in circulation that are not contained within cells^[2]^. ctDNA is used as a biomarker to identify specific mutations identified within a tumor that can guide treatment, and can also be used to monitor tumor progression^[3,4]^. Moreover, upwards of 70% of early-stage breast cancer patients have tumor-specific mutations present in ctDNA^[5]^. Genetic alterations present in a patient’s primary tumor biopsy can be detected in ctDNA in peripheral blood using sensitive mutation-targeted digital PCR methods^[6]^. Several studies have reported that detection of ctDNA in early stage breast cancer is associated with early recurrence, but further studies are needed to evaluate the use of ctDNA as an indicator of therapeutic response^[5,7,8]^. Recently, the Signatera™ personalized ctDNA test was shown to detect molecular indications of disease recurrence in breast cancer patients following adjuvant chemotherapy up to two years in advance of clinically detected metastatic relapse, with a sensitivity of 89% and a specificity of 100%^[9]^. Highlighting the potential impact of monitoring ctDNA for indications of disease recurrence, the Signatera™ test recently received a Breakthrough Device Designation from the FDA for use in detecting residual disease in patients with breast cancer, bladder cancer, non-small cell lung cancer, and colorectal cancer up to two years prior to conventional imaging methods. Furthermore, a method of personalized ctDNA detection, called “TARDIS”, which utilizes a novel targeted sequencing approach to evaluate tumor-specific mutations in ctDNA, was recently demonstrated to detect specific mutations in ctDNA as rare as 3 in 10^5[10]^. McDonald *et al*.^[10]^ used the TARDIS method to monitored patient-specific mutations in ctDNA in a breast cancer patient cohort throughout neoadjuvant treatment. This study demonstrated that breast cancer patients who achieved a pathological complete response exhibited significantly larger reductions in ctDNA, and had significantly lower ctDNA fractions, compared to patients with residual disease after neoadjuvant treatment. These studies demonstrate the utility of modern sequencing and PCR techniques to detect ctDNA as a molecular indicator of breast cancer, and indicate the potential that such methods have in monitoring therapeutic response and disease progression in breast cancer.

## Circulating tumor cells as prognostic biomarkers in breast cancer

In order for breast cancer to metastasize, breast cancer cells must escape the primary tumor site, enter into and travel through the circulation, exit the vasculature and invade into distant organ sites, and establish distant secondary lesions. CTCs are cancer cells that are found in circulation, and they provide predictive and prognostic information about a patient’s breast cancer. CTCs have been shown to have prognostic value in both metastatic and early breast cancer. In the context of metastatic breast cancer, high CTC counts are associated with poorer disease prognosis, and studies have shown that ~44% of patients with metastatic TNBC have ≥ 5 CTCs per 7.5 mL of blood, based on CellSearch®, the only currently FDA approved CTC enumeration platform^[11]^. In the context of early breast cancer, the SUCCESS trial demonstrated that the presence of CTCs at baseline prior to adjuvant chemotherapy, as well as the persistence of CTCs following adjuvant chemotherapy, was significantly associated with poorer disease free survival and worse overall survival in patients with early breast cancer^[12]^. A prospective study by Lucci *et al*.^[13]^ demonstrated that the detection of at least one CTC was prognostic of poorer progression-free survival among chemonaive patients with early stage breast cancer. Additional studies have demonstrated that the sustained presence of CTCs following neoadjuvant chemotherapy is associated with resistance to therapy^[14]^, and that persistent detection of CTCs in breast cancer patients following adjuvant therapy predicts poorer disease-free survival (DFS) and overall survival (OS)^[15]^. Furthermore, DFS and OS outcomes in patients whose CTCs disappeared after therapy were comparable to outcomes in patients lacking CTCs at baseline analysis^[15]^. These data indicate that changes in CTCs may be prognostic for patients with breast cancer, and suggest that interventions causing a durable reduction in CTCs lead to more favorable clinical outcomes for breast cancer patients. However, it remains unclear if reductions in the number of CTCs in response to treatment directly indicate a favorable response to a specific therapeutic regimen^[16,17]^.

Of great interest is the potential for CTCs to be used to guide intervention strategies and disease management for breast cancer. CTCs have been explored as biomarkers indicative of late breast cancer relapse. A study by Sparano *et al*.^[18]^ demonstrated that detection of CTC events five years after initial diagnosis was associated with a 13.1-fold higher risk of disease recurrence in patients with HER2-negative hormone receptor-positive breast cancer. A large retrospective pooled analysis of patients with metastatic breast cancer recently reported that the threshold of 5 CTCs per 7.5 mL blood is clinically relevant in stratifying patients with Stage IV disease as “Stage IV_indolent_” and “Stage IV_aggressive_”, where patients with Stage IV_indolent_ disease exhibited significantly longer overall survival times, regardless of breast cancer subtype, disease location, or prior treatment^[19]^. Recent data reported from the STIC CTC trial (a phase 3 non-inferiority trial wherein patients with metastatic ER+HER2- disease received hormone therapy or chemotherapy as *a priori* treatment choice, or were assigned to hormone therapy or chemotherapy based on CellSearch® CTC counts) indicate that not only was the treatment decision based on CTC count not inferior to the a priori treatment arm, but patients who were switched from hormone therapy to chemotherapy based on high CTC count had significantly longer progression free survival^[20]^. The STIC CTC trial is providing some of the first indications that CTC count can guide clinical action to not only assist with disease staging, but to improve outcomes for patients with metastatic breast cancer.

## Circulating proteins in plasma or serum are indicative of breast cancer progression and metastasis

Plasma proteins derived from both breast cancer cells and from the tumor microenvironment can be informative biomarkers of breast cancer progression and metastasis. Levels of carcinoembryonic antigen (CEA) and cancer antigen 15-5 (CA 15-3, MUC1) are independent prognostic markers for poor disease-free survival outcomes in patients with TNBC^[21]^. In patients with metastatic breast cancer, greater reductions in CA 15-3 levels during first-line chemotherapy significantly associated with improved time-to-progression and improved survival^[22]^. Furthermore, longitudinal increases in CA 15-3 and CEA may also provide early indications of breast cancer metastasis^[23,24]^. Cancer antigen 125 (CA 125) has been found to associate with the presence of pleural breast cancer metastasis^[25]^. Inclusion of CA 125 in a protein biomarker panel along with CA 15-3 demonstrated improved sensitivity to detect early breast cancer recurrence^[26]^. The utility of these proteins in the detection, response, and monitoring of breast cancer is achievable through minimally-invasive liquid biopsies, and can be performed in synchronization with assessment of other circulating biomarkers, such as ctDNA or CTCs.

## Non-epithelial CTCs, CTC clusters, and circulating cancer-associated cells

In breast cancer, CTCs have been defined in a variety of ways, most commonly based on the presence of EpCAM^[27]^ or epithelial cytokeratins^[28]^, and the absence of CD45^[27,28]^. However, CTCs lacking classical epithelial markers and have been also reported in patients with breast cancer^[29,30]^. CTCs with mesenchymal characteristics, including protein expression of vimentin^[31]^ and N-cadherin^[32]^, have been detected, demonstrating the phenotypic heterogeneity exhibited by breast cancer CTCs. Detection of CTCs expressing cancer stemness factors and undergoing epithelial-to-mesenchymal transition has been shown to indicate chemoresistance and poor clinical outcome in patients with metastatic breast cancer^[33]^. The emergence of breast cancer CTCs that do not fit within the widely accepted EpCAM-positive or epithelial cytokeratin-positive definitions demonstrates the importance of considering broader definitions of CTCs for better prognostic and predictive application of CTC enumeration.

The presence of CTCs in clusters with other CTCs (“homotypic clusters”) has also been reported, and is thought to indicate the viability and metastatic potential of CTCs. Preclinical models of cancer metastasis have demonstrated that CTCs in clusters, rather than individual CTCs, are more readily able to establish metastasis^[34,35]^. Additionally, the presence CTC clusters in patients with advanced stage breast cancer has been shown to be prognostic of poor outcome^[36-38]^. Exploration into the mechanisms of CTC clustering has shown that aggregation of CTCs in circulation occurs by homophilic CD44-CD44 interactions, and associates with advanced disease in preclinical mouse models. Furthermore, the presence of CD44+ CTC clusters in patients with metastatic breast cancer was found to significantly associate with poorer overall survival^[38,39]^.

In addition to circulating cancer cells, other circulating cancer-associated cells have been reported, including neutrophils^[40]^, macrophages^[41]^, and cancer-associated giant macrophage-like cells^[42]^. Preclinical models of breast cancer metastasis have shown that cells from the primary tumor stroma, including cancer associated fibroblasts (CAFs) can accompany breast cancer cells in circulation, ultimately facilitating metastatic dissemination^[43]^. These cells may circulate independently or in clusters with CTCs, and the significance of these circulating cancer-associated cells to breast cancer metastasis and the effects that they may have on treatment efficacy are of great interest.

## Cancer associated fibroblasts as a circulating biomarker in breast cancer

**Figure 1:**
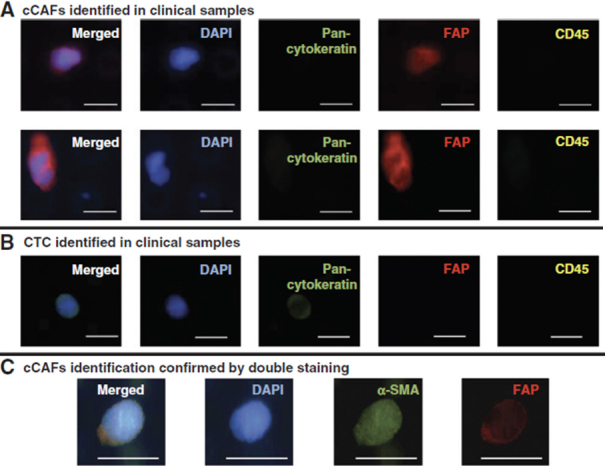
Identification of circulating cancer associated fibroblasts (cCAFs) in whole blood liquid biopsy samples from patients with metastatic breast cancer. A: cCAFs identified in clinical samples; B: circulating tumor cells (CTCs) identified in clinical samples; C: cCAFs identification confirmed by double staining. Scale bar = 20 µm. Data reproduced from Ao *et al*.^[46]^

**Figure 2:**
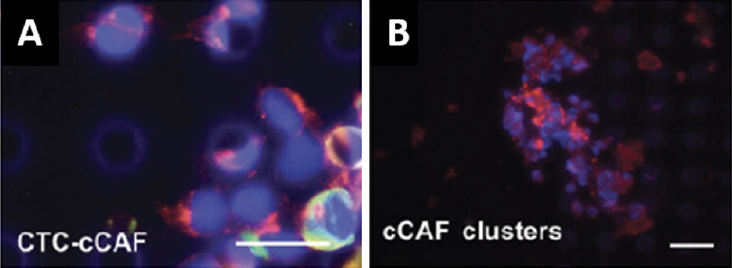
Clusters of circulating cells detected in whole blood liquid biopsy samples from a patient with metastatic breast cancer. A: representative heterotypic cluster of circulating tumor cells (CTCs) and circulating cancer associated fibroblasts (cCAFs); B: representative homotypic cluster of cCAFs. Blue: DAPI; Red: FAP; Green: Cytokeratin; White: CD45. Scale bar = 20 µm. Data reproduced from Ao *et al*.^[46]^

Our initial forays into liquid biopsy in breast cancer focused on the capture and enumeration of CTCs and the evaluation of serum and plasma protein biomarkers. In collaboration with Drs. Ram Datar and Richard Cote, we used a size-based microfiltration platform to capture circulating cells from liquid biopsy samples, and we identified those cells based on well-established markers using immunofluorescent staining. In addition to circulating breast cancer cells, we observed other cells that were negative for cytokeratin and negative for CD45. Prior observations by Duda *et al*.^[43]^ that CTCs can carry elements of the tumor microenvironment, including fibroblasts^[43]^, and reports by others of fibroblast-like cells in circulation of cancer patients^[44,45]^, suggested to us that these cells may be cancer associated fibroblasts. We found that many of these cells of indeterminate identity were positive for FAP and α-SMA (widely accepted markers for CAFs), and lacked cytokeratin expression or expression of the leukocyte common antigen, CD45, and we described this population of cells as circulating CAFs (cCAFs) [Figure 1]. Additionally, when isolated and put into culture, these cells exhibited characteristic CAF morphology. We reported that cCAFs are absent from healthy individuals, and are rarely seen in patients considered “cured” of breast cancer; i.e., minimum 5-years no evidence of disease. Of extreme interest to us, we discovered CTCs in circulating clusters with these cCAFs, as well as clusters containing only cCAFs [Figure 2], suggesting to us that these cCAFs are more than a putative cancer biomarker, and may be involved in mediating the metastatic process^[46]^. In an expanded cohort of breast cancer patients, we discovered that cCAFs and clusters of cCAFs with CTCs, which we describe as “heterotypic cCAF-CTC clusters” can be found in patients with earlier stage breast cancer (Sharma *et al*., unpublished data, manuscriopt pending review). The significance of these heterotypic cCAF-CTC clusters is not yet known, but we speculate may be informative for occult disseminated disease in early-stage breast cancer, and may serve as a novel prognostic biomarker.

To further evaluate the potential role of cCAFs in breast cancer metastasis, we developed a co-injection xenograft model, wherein human breast cancer cells are co-implanted with our primary human CAF cells. Consistent with previous reports, we observed not only increased primary tumor growth induced by co-injection of CAFs and cancer cells^[47,48]^, but increased disease metastasis (Sharma *et al*., unpublished data, manuscriopt pending review). In these xenograft models, we found human CAFs in circulation and present at sites of metastasis, confirming our notion that cCAFs represent CAFs from primary breast tumors that have entered the circulation. Additionally, we also observed clusters of circulating cells; these clusters often consisted of CTCs, cCAFs, or both CTCs and cCAFs, consistent with our observations from breast cancer patients. We further recapitulated these observations in syngeneic and spontaneous murine models of breast cancer metastasis. In addition, we observed cCAF/CTC heterotypic clusters far more often in models of metastatic breast cancer, compared to non-metastatic or poorly metastatic models. Our studies in these models show that the presence of cCAF/CTC heterotypic clusters is likely related to a breast cancer’s metastatic propensity.

## Goals and future directions of liquid biopsy in breast cancer

**Figure 3:**
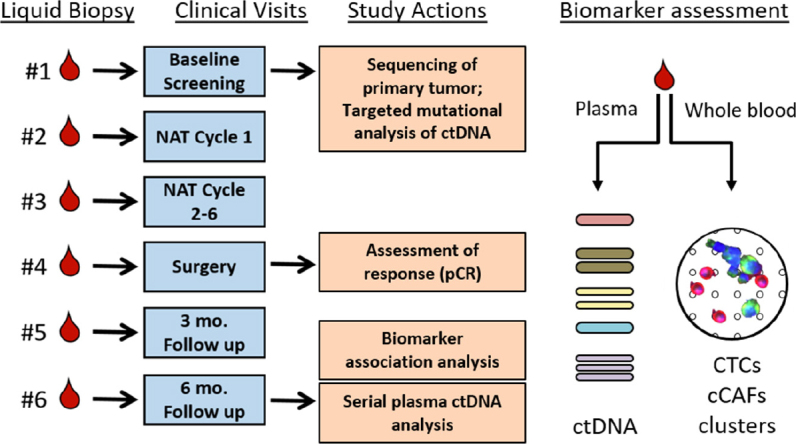
Schematic of study to simultaneously evaluate mutations in circulating cell-free tumor DNA (ctDNA), circulating tumor cells (CTCs), circulating cancer associated fibroblasts (cCAFs), and clusters of circulating cells in breast cancer patients undergoing neoadjuvant chemotherapy. Longitudinal assessment will determine if changes in these biomarkers associates with pathological complete response (pCR)

Our immediate goals are to advance the use of liquid biopsy to assess therapeutic responses in primary breast cancer and to monitor disease recurrence in the post-treatment setting. Moreover, a liquid biopsy approach that assesses genomic, cellular, and protein biomarkers could be expanded to sequence individual CTCs to correlate ctDNA data with clonal data directly obtained from CTC populations as well as sequence CTCs within heterotypic clusters. The compelling demonstration that assessment of circulating factors provides predictive and prognostic information of treatment efficacy would change the landscape of neoadjuvant and adjuvant therapy for breast cancer patients in a fundamental way: by identifying patients who are adequately treated and those who are not. There is an impetus to explore the use of real-time monitoring of circulating biomarkers to inform changes in therapeutic approach, to provide better care and identify additional treatment options for patients who may not initially respond to therapy. Our own observations and the rapid rise of ctDNA as an informative biomarker have led us to initiate a series of prospective liquid biopsy clinical studies in breast cancer patients to determine if longitudinal changes in circulating cellular biomarkers (CTCs, cCAFs, CTC homo/heterotypic clusters) and circulating cell-free tumor DNA are informative biomarkers of breast cancer response or resistance to neoadjuvant and adjuvant therapy (Study schematic, Figure 3). In these prospective studies, we will also collect plasma samples, enabling us to assess circulating protein markers or novel circulating DNA markers, to interrogate the utility of a more comprehensive liquid biopsy incorporating circulating cellular, protein, and genetic elements as a means of monitoring breast cancer treatment response in a minimally invasive manner. Our studies will complement other concurrent ongoing clinical trials to demonstrate the utility of liquid biopsy in monitoring disease progression and in therapeutic decision-making for breast cancer patients.

The potential for liquid biopsy methods to provide a minimally invasive means to detect breast cancer, predict treatment efficacy, and monitor disease recurrence is emphasized by recent efforts to encourage innovation and career training in the liquid biopsy space. New research is constantly improving on methods leading to improved sensitivity and specificity for circulating biomarkers in breast cancer detection. Novel biomarkers, including circulating microRNAs, novel populations of circulating cells, and circulating methylated DNA are being explored to compete with or complement existing circulating biomarkers to improve the utility of liquid biopsy for breast cancer care. Finally, the development of tumor-specific biomarker panels is advancing the use of liquid biopsy in personalized precision medicine. Liquid biopsy continues to represent a broad frontier for scientific discovery and a path of hope for patients with breast cancer and those at risk of developing breast cancer.

## Declarations

### Copyright

© The Author(s) 2019.
